# Insulin and glycolysis dependency of cardioprotection by nicotinamide riboside

**DOI:** 10.1007/s00395-024-01042-4

**Published:** 2024-03-25

**Authors:** Y. Xiao, Q. Wang, H. Zhang, R. Nederlof, D. Bakker, B. A. Siadari, M. W. Wesselink, B. Preckel, N. C. Weber, M. W. Hollmann, B. V. Schomakers, M. van Weeghel, C. J. Zuurbier

**Affiliations:** 1 Amsterdam UMC, Laboratory of Experimental Intensive Care and Anesthesiology, Department of Anesthesiology, University of Amsterdam, Meibergdreef 9, 1105 AZ Amsterdam, The Netherlands; 2 Amsterdam Cardiovascular Sciences Institute, Amsterdam, The Netherlands; 3 Department of Cardiology, Renmin Hospital of Wuhan University, Wuhan, 430060 People’s Republic of China; 4 Institut für Herz- und Kreislaufphysiologie, Medizinische fakultät und Universitätsklinikum Düsseldorf, Heinrich- Heine- Universität Düsseldorf, Düsseldorf, Germany; 5 Laboratory Genetic Metabolic Diseases, Location Academic Medical Center, Amsterdam University Medical Centers, University of Amsterdam, Amsterdam, The Netherlands; 6 Core Facility Metabolomics, Location Academic Medical Center, Amsterdam University Medical Centers, University of Amsterdam, Amsterdam, The Netherlands; 7 Amsterdam Gastroenterology Endocrinology and Metabolism Institute, Amsterdam, The Netherlands

**Keywords:** NAD^+^, Glycolysis, Insulin, PPP pathway, IR injury

## Abstract

Decreased nicotinamide adenine dinucleotide (NAD^+^) levels contribute to various pathologies such as ageing, diabetes, heart failure and ischemia–reperfusion injury (IRI). Nicotinamide riboside (NR) has emerged as a promising therapeutic NAD^+^ precursor due to efficient NAD^+^ elevation and was recently shown to be the only agent able to reduce cardiac IRI in models employing clinically relevant anesthesia. However, through which metabolic pathway(s) NR mediates IRI protection remains unknown. Furthermore, the influence of insulin, a known modulator of cardioprotective efficacy, on the protective effects of NR has not been investigated. Here, we used the isolated mouse heart allowing cardiac metabolic control to investigate: (1) whether NR can protect the isolated heart against IRI, (2) the metabolic pathways underlying NR-mediated protection, and (3) whether insulin abrogates NR protection. NR protection against cardiac IRI and effects on metabolic pathways employing metabolomics for determination of changes in metabolic intermediates, and ^13^C-glucose fluxomics for determination of metabolic pathway activities (glycolysis, pentose phosphate pathway (PPP) and mitochondrial/tricarboxylic acid cycle (TCA cycle) activities), were examined in isolated C57BL/6N mouse hearts perfused with either (a) glucose + fatty acids (FA) (“mild glycolysis group”), (b) lactate + pyruvate + FA (“no glycolysis group”), or (c) glucose + FA + insulin (“high glycolysis group”). NR increased cardiac NAD^+^ in all three metabolic groups. In glucose + FA perfused hearts, NR reduced IR injury, increased glycolytic intermediate phosphoenolpyruvate (PEP), TCA intermediate succinate and PPP intermediates ribose-5P (R5P) / sedoheptulose-7P (S7P), and was associated with activated glycolysis, without changes in TCA cycle or PPP activities. In the “no glycolysis” hearts, NR protection was lost, whereas NR still increased S7P. In the insulin hearts, glycolysis was largely accelerated, and NR protection abrogated. NR still increased PPP intermediates, with now high ^13^C-labeling of S7P, but NR was unable to increase metabolic pathway activities, including glycolysis. Protection by NR against IRI is only present in hearts with low glycolysis, and is associated with activation of glycolysis. When activation of glycolysis was prevented, through either examining “no glycolysis” hearts or “high glycolysis” hearts, NR protection was abolished. The data suggest that NR’s acute cardioprotective effects are mediated through glycolysis activation and are lost in the presence of insulin because of already elevated glycolysis.

**Supplementary Information:**

The online version contains supplementary material available at 10.1007/s00395-024-01042-4.

## Introduction

Nicotinamide adenine dinucleotide (NAD^+^) is a critical coenzyme and cofactor needed in many biochemical reactions in the cell. The biochemical functions of NAD^+^ and the physiological effects of NAD^+^ precursors impact cellular energetics, inflammation, metabolism and cell survival [50]. NAD^+^ metabolism is essential for maintaining normal physiological activities, and decreased NAD^+^ levels are related to various pathologies such as ageing, diabetes, metabolic diseases, cardiac diseases and ischemia–reperfusion (IR) injury [2, 6, 8]. Several NAD^+^ precursors, i.e., nicotinamide riboside (NR) and nicotinamide mononucleotide (NMN), are currently being tested in clinical trials directed at immunity, metabolic health, obesity, ageing and heart failure. Importantly, the US FDA recently excluded the use of NMN in dietary supplements, making NR the only commercially available NAD^+^ precursor. Here, we focus on the preclinical application of NR in acute cardiac IR injury to inform future therapeutic development of NR for patients with acute myocardial infarction.

NR has emerged as a leading NAD^+^ precursor candidate due to its high bioavailability, its safety in healthy adults [3, 31, 48] or obese patients [9], and its superiority in efficient NAD^+^ elevation in humans as compared to other agents [48]. We have recently demonstrated that NR, among several other potential cardioprotective compounds, was the only compound to retain cardioprotection in an in vivo IR model with clinically relevant anesthesia [53], suggestive of the potential of NR as a clinical cardioprotective strategy. However, underlying mechanisms of NR protection remains unexplored. It is also unknown whether NR can protect the isolated heart, which would indicate whether protection by NR is related to direct cardiac effects.

Both NMN and NAD^+^ administration have been reported to be cardioprotective in in vivo models of cardiac IR injury with exploration of the underlying mechanisms [34, 54, 57]. These NAD^+^ precursors may protect against cardiac IR through activation of sirtuins, autophagy, or the unfolded protein response (UPR) [23, 29, 43, 54]. Recently, an important role for the stimulation of glycolysis has been indicated for NMN protection against IR injury in isolated mouse hearts [34]. Glyceraldehyde-3-phosphate dehydrogenase (GAPDH) can control the rate of glycolysis and NAD^+^ is a necessary substrate in the GAPDH reaction, such that increases in NAD^+^ by NMN can possibly increase the cardiac glycolytic rate. It is known that activated glycolysis is a common cardioprotective mechanism of the heart against cardiac IR injury [58]. Because NMN is not commercially available anymore, NR now appears as optimal NAD^+^ precursor to mitigate acute cardiac IR injury. Thus, the first goal of the present study is to examine whether NR is protective in the isolated heart, and whether this is through activated glycolysis. Most importantly, insulin, when present already before ischemia, has been reported to abrogate cardioprotection for various cardioprotective interventions [38] such as IPC [16] or NHE1 inhibition [49]. Information is lacking whether the protective potential of NAD^+^ precursors is also dependent on the presence of insulin. Such information is especially important for translation to the clinical condition, where insulin therapy is often provided due to the presence of stress hyperglycemia during cardiac surgery or acute myocardial infarction condition. Therefore, in the present study, we addressed several questions in relation to NR cardioprotection: (1) Can NR protect the isolated heart against IR injury? (2) Is cardioprotection by NR due to the activation of glycolysis or other metabolic pathways and (3) Is NR protection dependent on the presence of insulin?

## Method

### Animals

This study was conducted in accordance with the *Guide for the Care and Use of Laboratory Animals* [1], published by the National research Council (8th edition, 2011), and protocols were registered and approved by the Animal Ethics Committee of the Academic Medical Center, Amsterdam, The Netherlands. For acclimatization, C57BL6/N adult male mice (Charles River, Lyon, France) were housed for at least 1 week in standard housing conditions in the Animal Research Institute AMC (ARIA) before experiment. Mice were fed with lab diet and given water ad libitum.

Although sex differences in tolerance to IR injury are not always observed in preclinical models [5], other studies have reported that female sex offer protection against IR injury possible due to a protective role of estrogen [33, 40]. Therefore, to reduce the chance of higher variance in our data and number of animals needed for this study, we have focused only on male mice.

### Isolated heart perfusion and functional measurement

The preparations for intact mouse heart have been described in our previous study [49]. In brief, male mice weighing 25.5 ± 2.2 g (11–15 weeks old) were anesthetized with Fentanyl (0.5 mg/kg), Midazolam (9.4 mg/kg), Acepromazine (9.4 mg/kg) and heparinized (15 IU) by intraperitoneal injection. Mice were intratracheally ventilated with 50% O_2_ and 50% N_2_ after adequacy depth of anesthesia was ensured by loss of pedal withdrawal reflex. Then, the aorta was cannulated in the chest, afterwards the heart was excised and connected to the Langendorff setup immediately. Hearts were perfused with standard Krebs–Henseleit (KH) buffer, which consisted of (mmol/L) 118 NaCl, 4.7 KCl, 1.2 MgSO_4_, 1.2 KH_2_PO_4_, 25 NaHCO_3_, 0.5 EDTA, 2.50 CaCl_2_. 5.5 mM glucose (Glu) and 1% (g/L) albumin—0.2mM free fatty acid (FFA) palmitate (Glu + FFA) were added to the KH buffer as substrates. Substrates were altered as described in the experimental protocols for mechanisms exploring. A water thermo-regulator was used to maintain constant temperature (37 ± 0.5 °C) for the whole Langendorff system, KH buffer was filtered (0.45µm) and gassed with 95% O_2_ / 5% CO_2_ to obtain a pO_2_ ≥ 600 mmHg and a pH = 7.4.

Cardiac function was assessed by a water-filled balloon inserted into left ventricle and connected to a pressure transducer. 20 min equilibration was allowed to all hearts. The water volume in the balloon was adjusted to obtain an initial end diastolic pressure (EDP) of 3–9 mmHg, coronary flow (< 4 ml/min) was adjusted to set the initial perfusion pressure at 90 ± 5 mmHg. Hearts were excluded, a priori, when developed left ventricular pressure (DLVP, DLVP = systolic pressure- EDP) was below 65 mmHg, and/or heart rate (HR) was below 280 beats per minute (bpm), and/or the hearts developed irregular heartbeat after 20 min stabilization. Rate pressure product (RPP) was calculated from DLVP × HR. Time of contracture (TOC) was defined as the time from the start of ischemia when EDP progressed above 3.0 mmHg, which represents the time that ischemic anaerobic glycolysis stops and ΔG_ATP_ falls below the level needed to support ion pumps and cross-bridges detachment [32]. Thus, TOC is an indirect measurement of anaerobic glycolysis activity during ischemia, prolonged TOC suggesting longer glycolysis activation during ischemia and vice versa.

### Langendorff protocols to determine IR injury

**Fig. 1:**
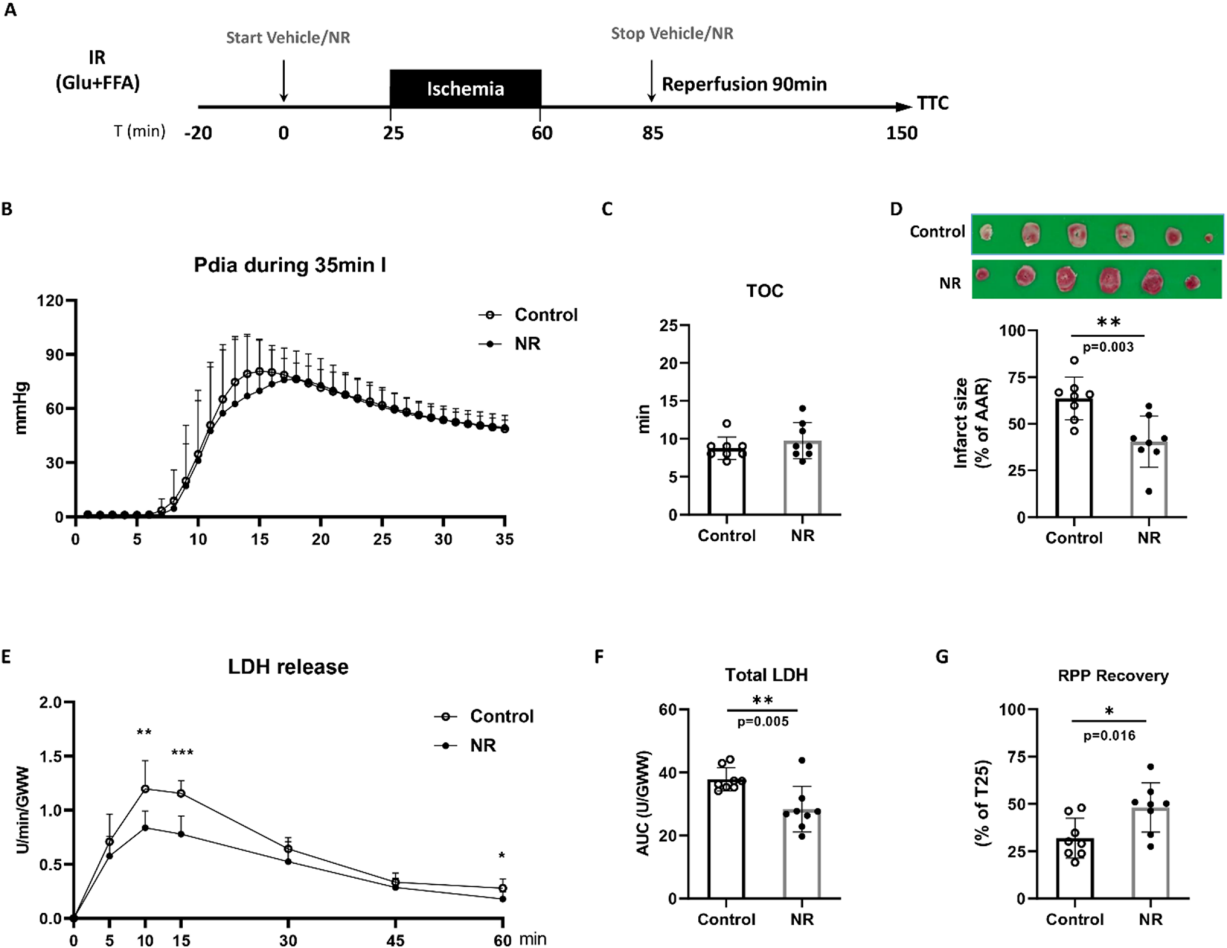
NR protected against cardiac IR injury in low glycolysis condition. **A** Hearts were perfused with glucose and free fatty acid (Glu + FFA). After 25 min baseline, hearts were subjected to 35 min ischemia followed by 90 min reperfusion, along with administration of vehicle or NR (50 mg/L); **B** End-diastolic pressure development during ischemia; **C** Time to contracture (TOC), the time until when end-diastolic pressure raises above 3 mmHg; **D** Image of TTC staining (upper panel) and quantified infarct size related to AAR (lower panel); **E** LDH release at different time points during reperfusion, normalized to coronary flow and heart wet weight; **F** Total LDH release during reperfusion, normalized to coronary flow and heart wet weight; **G** RPP recovery at the end of reperfusion relative to baseline T = 25 min. *P < 0.05, **P < 0.01, ***P < 0.001 vs Control group

**Fig. 2:**
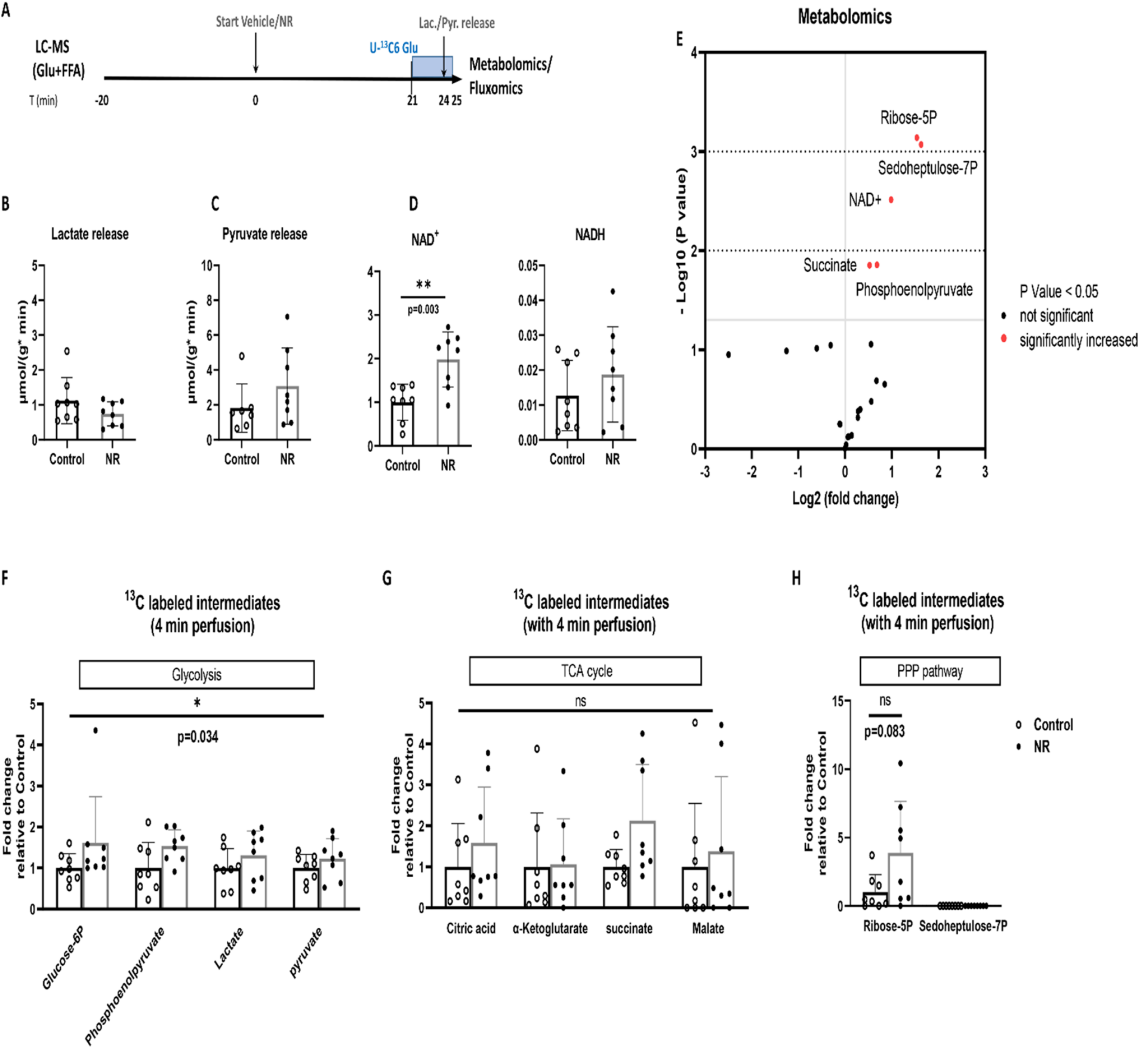
NR increased NAD^+^, intermediates of glycolysis, PPP and TCA, and activated glycolysis in low glycolysis hearts. **A** Hearts were perfused with glucose and free fatty acid (Glu + FFA) for 25 min with vehicle or NR (50 mg/L) administration. During the last 4 min, unlabeled glucose was replaced with isotope labeled [U-^13^C6] glucose; **B** Lactate release measured in coronary effluent; **C** Pyruvate release measured in coronary effluent (one value was missing in control group due to undetectable low pyruvate concentration); **D** NAD^+^ and NADH content measured in freeze-dried heart tissues by LC–MS; **E** Total metabolites (unlabeled and labeled) were determined by LC–MS techniques in freeze-dried hearts tissues; **F** ^13^C-glucose labeling of glycolytic intermediates during a 4 min period of [U-^13^C6] glucose perfusion; **G** ^13^C-glucose labeling of TCA cycle intermediates during a 4 min period of [U-^13^C6] glucose perfusion; **H** ^13^C-glucose labeling of PPP pathway intermediates during a 4 min period of [U-^13^C6] glucose perfusion. *P < 0.05, **P < 0.01 vs Control group

**Fig. 3:**
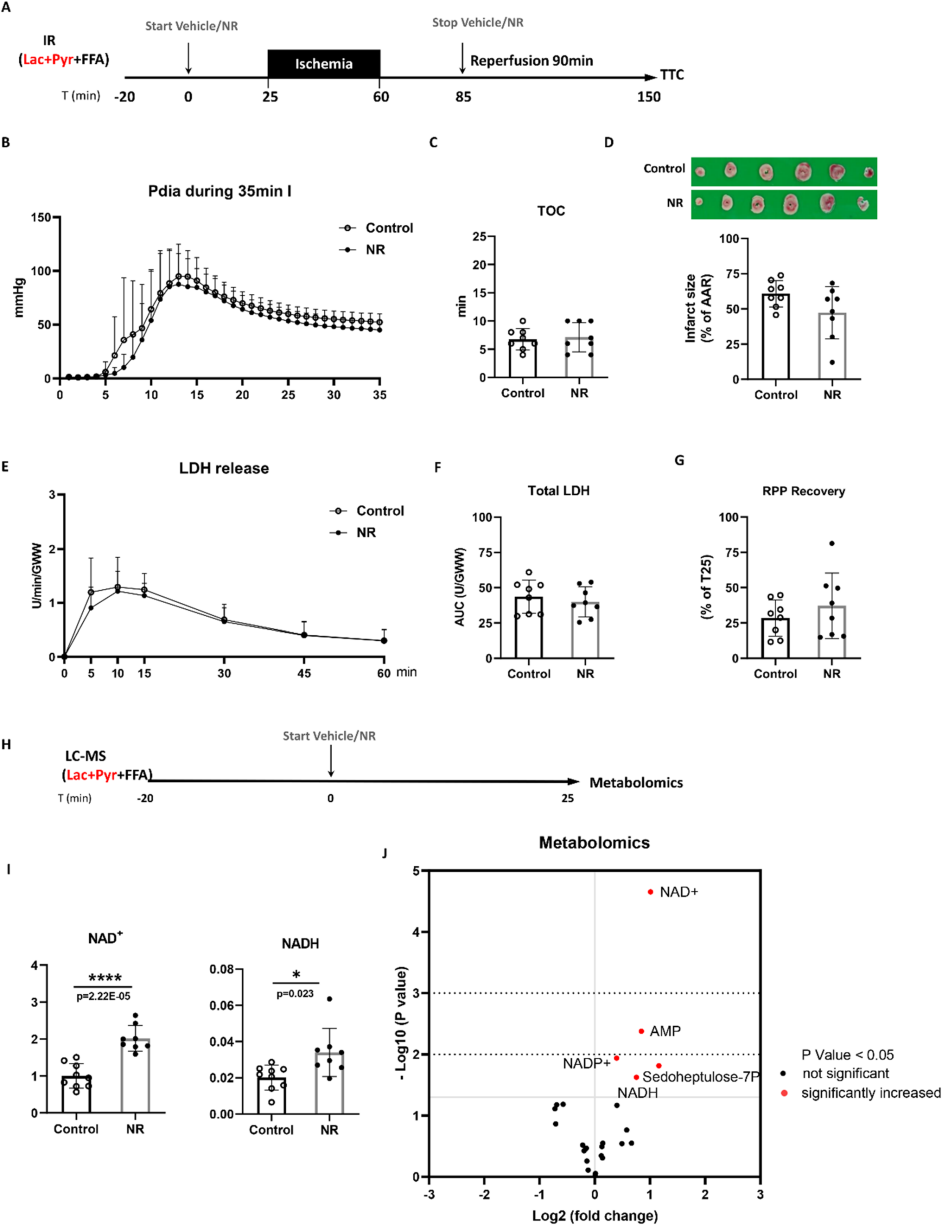
NR lost protection in no glycolysis condition. **A** Hearts were perfused with lactate, pyruvate, and free fatty acid (Lac + Pyr + FFA). After 25 min baseline, hearts were subjected to 35 min ischemia followed by 90 min reperfusion, along with administration of vehicle or NR (50 mg/L); **B** End-diastolic pressure development during ischemia; **C** Time to contracture (TOC); **D** Image of TTC staining (upper panel) and quantified infarct size related to AAR (lower panel); **E** LDH release at different time points during reperfusion, normalized to coronary flow and heart wet weight; **F** Total LDH release during reperfusion, normalized to coronary flow and heart wet weight; **G** RPP recovery at the end of reperfusion relative to baseline T = 25 min. **H** Hearts were perfused with lactate, pyruvate, and free fatty acid (Lac + Pyr + FFA) for 25 min with vehicle or NR (50 mg/L) administration. **I** NAD^+^ and NADH content measured in freeze-dried heart tissues by LC–MS; **J** Total metabolites were determined by LC–MS techniques in freeze-dried hearts tissues. ****P < 0.0001 vs Control group

**Fig. 4:**
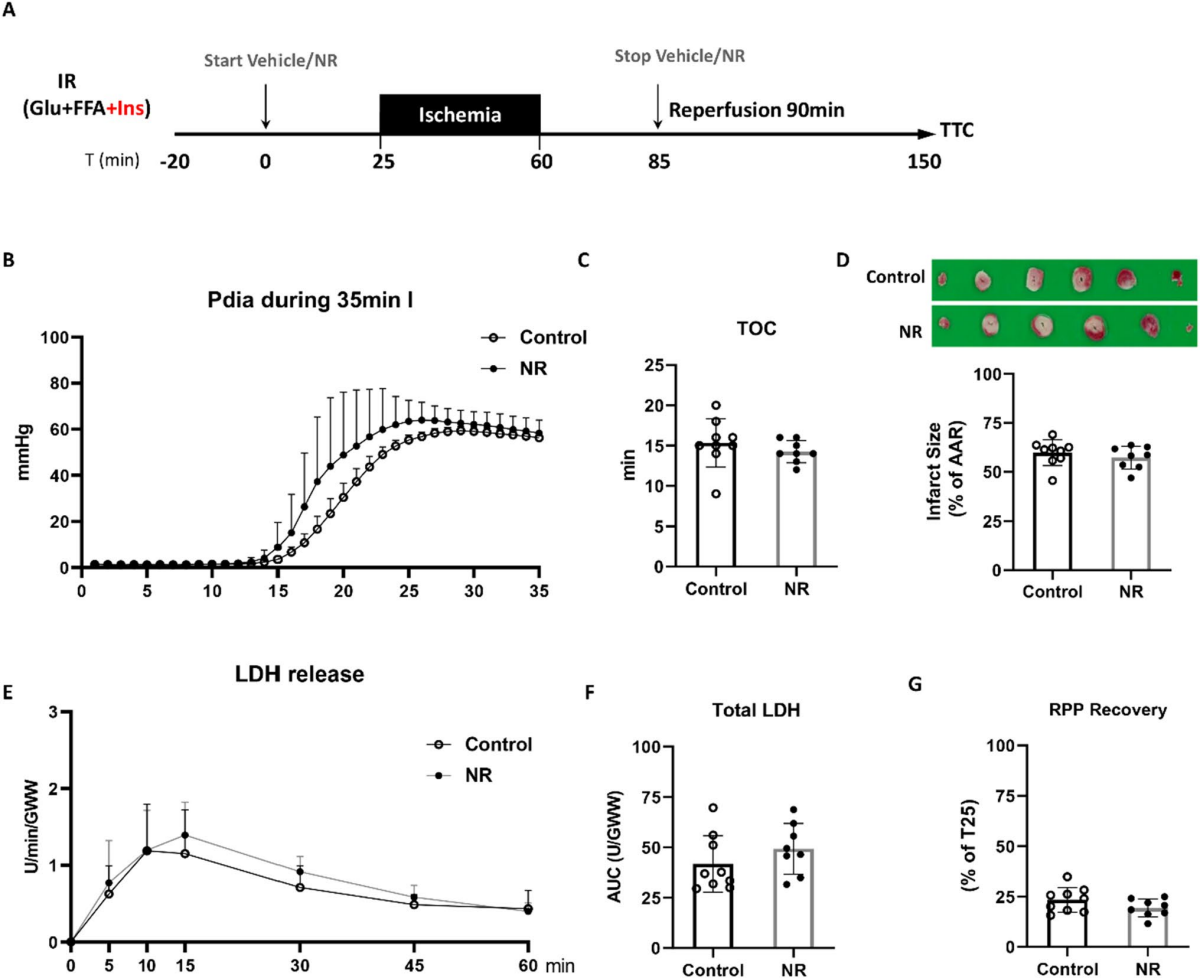
NR lost protection in high glycolysis condition. **A** Hearts were perfused with glucose, free fatty acid, and insulin (Glu + FFA + Ins). After 25 min baseline, hearts were subjected to 35 min ischemia followed by 90 min reperfusion, along with administration of vehicle or NR (50 mg/L); **B** End-diastolic pressure development during ischemia; **C** Time to contracture (TOC); **D** Image of TTC staining (upper panel) and quantified infarct size related to AAR (lower panel); **E** LDH release at different time points during reperfusion, normalized to coronary flow and heart wet weight; **F** Total LDH release during reperfusion, normalized to coronary flow and heart wet weight; **G** RPP recovery at the end of reperfusion relative to baseline T = 25 min

The dosage of NR (50 mg/L) was chosen based on previous in vivo rat study, which showed protection against IR injury [53]. 50 mg/L NR (HY-123033A, Med Chem Express) or vehicle (KH buffer) was administrated following the IR protocol described below. We first wanted to observe whether NR can protect the heart from IR injury: following 20 min equilibration, hearts were subjected to 25 min baseline, 35min global ischemia and 90min reperfusion with Glu + FFA perfusion (Fig. 1A). Treatments (NR or vehicle administration) were infused during 25 min prior to ischemia and the first 25 min of reperfusion through a side port above the aortic cannula at 1% of coronary flow (Fig. 2). To further explore whether NR protects through activation of glycolysis, separate metabolic groups with identical protocols as described above, were examined: 2) a “no glycolysis” series, replacing glucose in the perfusate with 1 mM lactate (Lac) and 0.1 mM pyruvate (Pyr) (Lac + Pry + FFA) (Fig. 3A), and 3) a “high glycolysis” series with insulin present during the whole perfusion protocol, adding 30 mU/L insulin (Ins) to the perfusate (Glu + FFA + Ins (Fig. 4A). Finally, an additional series was performed where insulin was only present during the reperfusion period, to examine whether insulin reduce cardiac IR injury when administered only at reperfusion. All animals were randomized to morning/afternoon to ensure we had the same time-of-day distribution in all groups and avoid the influence of circadian rhythm on IR tolerance [10].

### TTC staining

At the end of 90 min reperfusion, heart was weighed and then quickly frozen at -20 °C. For 2,3,5-triphenyltetrazolium chloride (TTC, Sigma-Aldrich, St. Louis, MO, USA) staining, the heart was cut into 5–6 slides which around 1 mm thickness and then incubated in 1% TTC buffer (pH = 7.4) at 37 °C for 20 min on the shaker at 300 rpm. Then, the heart slices were incubated in 4% formaldehyde for 2 h at room temperature in the dark. The heart slices were then scanned and infarct size (%IS, percentage of infarct size relative to area at risk) was quantified blindly with SigmaScan Pro5 software.

### LDH measurement

The coronary effluent was collected at 5, 10, 15, 30, 45 and 60 min of reperfusion. Lactate dehydrogenase (LDH) was measured by spectrophotometry at 340 nm, pH = 7.5 and 25 °C blindly. Briefly, activity was determined by monitoring the rate of NADH oxidation in assay buffer (containing 50 mM pyruvate as substrate) for 4 min. The Total LDH release was determined by calculating the area under the curve of LDH release. The LDH activity was normalized to actual coronary flow and heart wet weight.

### Langendorff protocols for metabolomics and fluxomics studies employing LC–MS

**Fig. 5:**
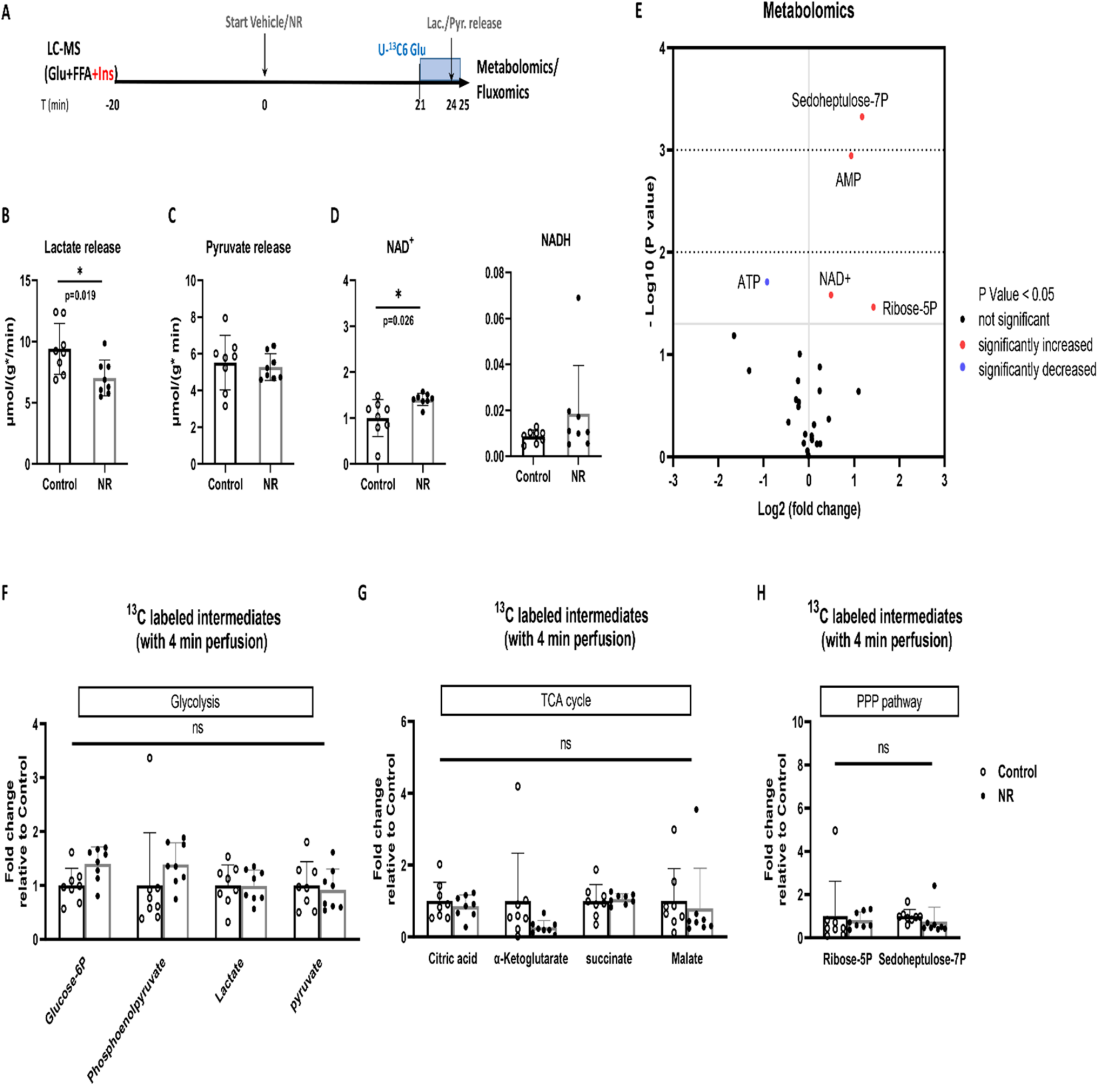
NR increased NAD^+^ and PPP intermediates without affecting glycolysis in insulin-treated, high glycolysis, hearts. **A** Hearts were perfused with glucose, free fatty acid, and insulin (Glu + FFA + Ins) for 25 min with vehicle or NR (50 mg/L) administration. During the last 4 min, glucose was replaced with isotope labeled [U-^13^C6] glucose; **B** Lactate release measured in coronary effluent; **C** Pyruvate release measured in coronary effluent; **D** NAD^+^ and NADH content measured in freeze-dried heart tissues by LC–MS; **E** Total metabolites (unlabeled and labeled) were determined by LC–MS techniques in freeze-dried hearts tissues; **F** ^13^C-glucose labeling of glycolytic intermediates during a 4 min period of [U-^13^C6] glucose perfusion; **G** ^13^C-glucose labeling of TCA cycle intermediates during a 4 min period of [U-^13^C6] glucose perfusion; **H** ^13^C-glucose labeling of PPP pathway intermediates during a 4 min period of [U-^13^C6] glucose perfusion. *P < 0.05 vs Control group

Hearts were excised as mentioned above for the IR studies, and perfused in Langendorff setup under constant flow (2.5 ml/min) with for the three metabolic series: (1) low glycolysis (Glu + FFA) (Fig. 2A), (2) no glycolysis (Fig. 3H) or (3) insulin, high glycolysis (Glu + FFA + Ins) (Fig. 5A). Following 20 min equilibration, hearts were subjected to 25 min baseline perfusion along with either vehicle or NR delivery. For glycolytic flux measurements, 5.5 mM glucose in KH buffer was replaced with 5.5 mM isotope labeled [U-^13^C6] glucose (initial molar enrichment (MPE): 99%; Cambridge Isotope Laboratories, Andover, USA) for the last 4 min. of the 25 min baseline perfusion period. Then, coronary effluent was collected and each heart was freeze-clamped in liquid N_2_. Hearts were then freeze-dried and cardiac metabolites were extracted for LC–MS/MS based metabolomics and fluxomics analysis in a blinded fashion.

Metabolomics was performed as previously described, with minor adjustments [41, 56]. Samples were freeze-dried, crunched and approximately 2 mg weighted in a 2 ml tube. A 75 µl mixture of internal standard adenosine-^15^N_5_-monophosphate (100 µM) was added to each sample. Subsequently, 425 µl water, 500 µl methanol, 1 ml chloroform and a 5 mm Qiagen Stainless Steel Bead were added. Using a Qiagen TissueLyser II, samples were homogenized at 30 times/second. Samples were then centrifuged for 10 min at 14.000 rpm. The top layer, containing the polar phase, was transferred to a new 1.5 ml tube and dried using a vacuum concentrator at 60°C. Dried samples were reconstituted in 100 µl methanol/water (6/4; v/v). Metabolites were analyzed using a Waters Acquity ultra-high-performance liquid chromatography system coupled to a Bruker Impact II™ Ultra-High Resolution Qq-Time-Of-Flight mass spectrometer. Samples were kept at 12 °C during analysis and 5 µl of each sample was injected. Chromatographic separation was achieved using a Merck Millipore SeQuant ZIC-cHILIC column (PEEK 100 × 2.1 mm, 3 µm particle size). Column temperature was held at 30 °C. Mobile phase consisted of (A) 1:9 acetonitrile:water and (B) 9:1 acetonitrile:water, both containing 5 mM ammonium acetate. Using a flow rate of 0.25 ml/min, the LC gradient consisted of: Dwell at 100% Solvent B, 0–2 min; Ramp to 54% Solvent B at 13.5 min; Ramp to 0% Solvent B at 13.51 min; Dwell at 0% Solvent B, 13.51–19 min; Ramp to 100% B at 19.01 min; Dwell at 100% Solvent B, 19.01–19.5 min. Mass spectrometry (MS) data were acquired using negative and positive ionization in full scan mode over the range of m/z 50–1200. Data were analyzed using Bruker TASQ software version 2021.

For fluxomics, isotope ratios, tracer purity and correction for the contribution of naturally occurring isotopes were calculated using IsoCorrectoR [21]. For metabolomics, summed peak areas of all isotopes were used, normalized to the internal standard and heart weight. Metabolites amounts are thus given as relative amounts to the one internal standard, and not as absolute amounts because internal standards specific for each metabolite measured were not used. Metabolite identification has been based on a combination of accurate mass, (relative) retention times and fragmentation spectra, compared to the analysis of a library of standards.

### Lactate/pyruvate measurement

Coronary effluent was used for detection of lactate and pyruvate release for the experimental series where glucose was present in the buffer. For the lactate measurement, 40 µL effluent was added in 200 µL assay mix (contains 0.4 M glycine, 0.4M hydrazine hydrate and 0.38 mM NAD^+^, pH = 9.5), and absorbance was read as reference. After 10 µL LDH (568 U/ml) was added, the NADH production was monitored for 60min at 340 nm and 25 °C. For the pyruvate measurement, 80 µL effluent was added in 200 µL assay buffer (contains 0.5 M KH_2_PO_4_, 10% Triton-X100, and 0.17 mM NADH, pH = 7.5), and absorbance was read as reference. After 10 µL LDH (1136 U/ml) was added, the NADH production was monitored for 90min at 340 nm and 25 °C. Lactate and pyruvate concentration were calculated based on the standard curve, and normalized to heart dry wet (mg) and flow (ml/min).

### Statistical analysis

Results are expressed as mean ± SD, n is the number of animals used unless stated otherwise. For the IR experiments, an initial *n* = 8 was determined to be able to detect a clinically relevant decrease of 25% in infarct size with an α = 0.05, SD = 9, and a power of 90%. For the LC–MS measurements, an initial *n* = 8 was determined from previous study [55] to be able to detect a clinically relevant change of 25% in glycolytic intermediates with an α = 0.05, SD = 11.5, and a power of 90%. Shapiro–Wilk was used to test the normality distribution of data. Possible outliers were identified using Grubbs’s test. Unpaired *t* test was performed when data were normally distributed, non-normally distributed data was analyzed with Independent-Samples Kruskal–Wallis test, unless otherwise stated. For metabolic pathways we first depict NR effects on the detectable metabolites from glycolysis, TCA cycle, PPP pathway and the NAD + pathway using a volcano plot using uncorrected *P* values; for *P* values < 0.05, false discovery rates (FDR) and variable importance projection (VIP) scores are also provided in the text, and for each variable in the supplementary files. Then we specifically tested four a priory determined hypotheses whether NR altered (1) NAD^+^, and pathway activities of (2) glycolysis, (3) TCA and (4) PPP. The pathways were represented by more than one intermediate metabolite (i.e., glycolysis, TCA cycle and pentose phosphate pathway). Pathway activities were determined by examining as repeated measure, the absolute amount of ^13^C-glucose label measured in the pathway intermediates, and tested whether there was an overall significant NR effect by employing two-way repeated measurement ANOVA. Statistics and figures were conducted using IBM SPSS statistics version 26 (International Business Machines Corp., Armond, NY, USA) and GraphPad Prism 8.0 (GraphPad Software, Inc., La Jolla, CA, USA). In all tests, significance was accepted for *P* < 0.05.

All the original data was deposited on Figshare (https://figshare.com/s/bdc5d4747f7894f9c78b).

## Results

### NR treatment reduces IR induced cardiac injury in the mouse heart

No differences existed in the Initial cardiac function between our treatment groups (suppl. Table 1). Using an intact heart IR model perfused with glucose and free fatty acid (Fig. 1A), we observed that compared with the control group, NR treatment reduced IR induced cell death: infarct size (expressed as percent of area at risk) was significantly decreased by ≈ 40% in NR group (Fig. 1D); Total LDH release was significantly suppressed by ≈ 25% in NR treated hearts compared with control hearts (Fig. 1E and F). Consistent with less cell death, hearts in the NR group exhibited better post-ischemic recovery: rate pressure product (RPP) recovery (expressed as percent of pre-ischemic RPP) increased by ≈ 50% in NR group (Fig. 1G) compared to the control group. No difference of TOC was observed (Fig. 1B and C), indicative that NR did not affect ischemic anaerobic glycolysis. Other cardiac mechanical parameters (DLVP, + dp/dt, − dp/dt) were monitored throughout the experiments and showed no difference between groups (Suppl. Figure 1).

### NR increased NAD^+^, intermediates of glycolysis, PPP and TCA, and activated glycolysis in glucose + FA perfused hearts

To evaluate metabolic effects of 25 min NR perfusion, heart effluent was collected to measure lactate and pyruvate release as proxy for anaerobic glycolysis activity, total metabolites (unlabeled and labeled) were determined by LC–MS techniques in freeze-clamp hearts to detect levels of metabolic intermediates, and metabolic activity of pathways determined by examining ^13^C-glucose labeling of metabolic intermediates during a 4 min period of ^13^C-glucose infusion (Fig. 2A). There was no difference in cardiac function between these treatment groups analysed for metabolic characteristics (suppl. Table 2).

NR was without effect on anaerobic glycolysis, as reflected by similar amounts of lactate (*P* = 0.382) and pyruvate (*P* = 0.152) release from control and NR-treated hearts (Fig. 2B–C).

NR perfusion (25 min) increased cardiac NAD^+^ content by almost a factor of two, without affecting NADH (Fig. 2D). NR increased the metabolic intermediates phosphoenolpyruvate (control 0.125 ± 0.057; NR 0.201 ± 0.050 *P* = 0.014, FDR = 0.067, VIP score = 1.36), the pentose phosphate pathway (PPP) intermediates of ribose-5P [R5P, *P* = 0.001, FDR = 0.010, VIP score = 1.73)] and sedoheptulose-7P [S7P, *P* = 0.001, FDR = 0.010, VIP score = 1.79)] by a factor of three, and the tricarboxylic acid (TCA) cycle intermediate succinate [*P* = 0.014, FDR = 0.067, VIP score = 1.33)] (Fig. 2E). All metabolomics data for this series are provided in data file on Figshare (see above for link).

Because levels of metabolic intermediates do not inform about pathway flux, we employed the inflow of ^13^C-glucose labeling into metabolic intermediates during 4 min infusion with ^13^C-glucose. This treatment demonstrated that NR significantly increased glycolysis by approximately 42% as indicated by increased ^13^C labeled glycolysis intermediates (*P* = 0.034, Fig. 2F). NR also showed a non-significant trend to increase PPP activity, as reflected by 3.9 times increased labeling of R5P (*P* = 0.083, Fig. 2H). Remarkable, there was no ^13^C glucose going into S7P, reflected by zero labeling of S7P. The observed NR-induced increase in S7P amount is thus probably due to an increased conversion of elevated NAD^+^ into ribose-5P by the NADase CD38 enzyme, with ribose-5P then feeding the oxidative PPP to generate S7P. NR treatment did not affect the overall TCA cycle pathway flux (*P* = 0.275, Fig. 2G).

### NR-induced cardioprotection is vanished with no glycolysis

To explore whether glycolysis is necessary for NR cardioprotection, glycolysis was bypassed by replacing glucose in the perfusate by lactate and pyruvate (Fig. 3A). Under these conditions, NR-induced cardiac protection was abolished, as indicated by equal level of infarct size (Fig. 3D), LDH release (Fig. 3E and F) and RPP recovery (Fig. 3G) between NR and control group. The loss of glycolysis reduced TOC relative to glucose-perfused hearts (from 8.8 to 6.8 min, *P* = 0.035), indicative of less glycogen and decreased anaerobic glycolysis under these conditions; however, TOC was unaltered by NR (Fig. 3B and C). In addition, NR did not affect other cardiac mechanical parameters (DLVP, + dp/dt, − dp/dt) (Suppl. Figure 2).

NAD^+^ and NADH content and metabolic pathways in the absence of glucose were evaluated (Fig. 3H). There was no difference of cardiac function between two groups (suppl. Table 2). The effect of NR on elevating NAD^+^ content (Fig. 3I) and PPP intermediate (*P* = 0.015, FDR = 0.095, VIP score = 1.48 for S7P; Fig. 3J) was still present in the absence of glucose. NR also increased other intermediates of the NAD^+^ pathway (*P* = 0.024, FDR = 0.118, VIP score = 1.41 for NADH (Fig. 3i), *P* = 0.012, FDR = 0.095, VIP score = 1.44 for NADP^+^; Fig. 3J). NR did not increase glycolysis or TCA metabolic intermediates. NR also increased AMP in no glycolysis hearts (*P* = 0.004, FDR = 0.052, VIP score = 1.63 Fig. 3J). All metabolomics data of the no glucose series is provided in data file on Figshare (see link above).

### NR-induced cardioprotection is abrogated in the presence of insulin

To explore whether NR is still protective in the presence of insulin, and thus in conditions of highly activated glycolysis, insulin was added to the perfusate throughout the whole IR protocol (Fig. 4A). TOC was equal between the control and NR group (Fig. 4B and C), but insulin largely delayed TOC compared to the glucose-perfused, low glycolysis group (going from 8.8 min to 15.4 min, *P* = 3.70E-05), indicative of increased glucose uptake and thus glycogen accumulation and anaerobic glycolysis during ischemia in the presence of insulin. No cardioprotective effect of NR was observed in insulin-perfused hearts, as demonstrated by equal levels of infarct size (Fig. 4D), LDH release (Fig. 4E and F), and RPP recovery (Fig. 4G) between NR and control group. NR was also without effect on DLVP, + dp/dt, − dp/dt (Suppl. Figure 3).

### NR increased NAD^+^ and PPP intermediates without affecting glycolysis in insulin-treated, high glycolysis, hearts

We then evaluated glycolytic parameters, NAD^+^ and NADH content and metabolic pathways in the presence of insulin (Fig. 5A). There was no difference in cardiac function between the treatment groups (suppl. Table 2). Insulin increased lactate release by a factor of 8 as compared to the low glycolysis hearts, indicating insulin’s activating effect on glycolysis in isolated mouse hearts (Fig. 2B versus Fig. 5B). In these high glycolysis hearts, NR now decreased lactate release (control 9.40 ± 2.08 µmol/g* min; NR 7.03 ± 1.47 µmol/g* min, *P* = 0.019, Fig. 5B), without affecting pyruvate release (*P* = 0.685, Fig. 5C), suggestive of decreased anaerobic glycolysis with NR in the presence of high glycolysis.

Insulin did not block NR’s effects on elevating NAD^+^ content (Fig. 5D) and PPP intermediates (*P* = 0.034, FDR = 0.185, VIP score = 1.53 for R5P, *P* = 0.0005, FDR = 0.012, VIP score = 2.16 for S7P; Fig. 5E), although no significant increase in NADH was observed. NR did not increase glycolysis or TCA metabolic intermediates. Surprisingly, NR increased AMP and decreased ATP in high glycolysis hearts (ATP: control 0.322 ± 0.133; NR 0.170 ± 0.090 *P* = 0.019, FDR = 0.175, VIP score = 1.53: AMP: control 0.996 ± 0.377; NR 0.907 ± 0.494 *P* = 0.001, FDR = 0.015, VIP score = 1.96, Fig. 5E), suggestive of NR being detrimental to the cardiac energy status in insulin-treated, high glycolysis hearts. All metabolomics data of the insulin series is provided in data file on Figshare (see link above).

The flux measurements with ^13^C-glucose demonstrated that NR loses its activating effect on glycolysis in the presence of insulin (*P* = 0.207, Fig. 5F). NR also had no effects on activity of the PPP pathway (*P* = 0.466, Fig. 5H) or the TCA cycle pathway (*P* = 0.310, Fig. 5G). However, with insulin present, ^13^C-labeling of S7P was now observed, indicating that insulin is necessary to feed glucose into S7P.

### Insulin administration only at reperfusion is cardioprotective

A separate series of experiments was performed in (glucose + FFA) perfused mouse hearts, to examine whether the administration of insulin only at reperfusion is protective in our isolated heart model. These experiments clearly show the cardioprotective effects of insulin when administered at start of reperfusion only (Suppl. Figure 4).

## Discussion

The major novel finding of the present study is that the NAD^+^ precursor NR cannot protect the isolated heart against IR injury or activate glycolysis in insulin-stimulated, high glycolysis, hearts. Additional important findings are that (1) the NAD^+^ precursor NR can protect the isolated heart against acute IR injury, (2) NR’s protection is associated with an activation of glycolysis, (3) NR cannot protect hearts without glycolysis, suggesting that NR protection is through glycolysis, (4) short-term NR perfusion increases cardiac NAD^+^ and PPP intermediates, and finally (5) NR reduces lactate release and ATP content and increases AMP content in insulin-stimulated, high glycolysis hearts, indicative that NR impairs energy metabolism in high glycolysis, insulin-perfused hearts. These data suggest that NR can reduce cardiac IR injury through glycolysis activation, but only in hearts that have relatively moderate glycolytic rates.

### NR, glycolysis and protection

The NAD^+^/NADH ratio regulates metabolic pathways such as glycolysis and oxidative phosphorylation in the mitochondria. Although the protective effects of NAD^+^ precursors have been mostly ascribed to de-acetylation by sirtuin activation [23], it has recently been shown that NMN reduced cardiac IR injury of isolated mouse hearts, primarily through the activation of glycolysis [34]. NR and NMN are both NAD^+^ precursors, but their distribution, metabolism and compartmentation are different [6, 8, 23], and it was thus unknown whether NR would constitute similar protective effects as NMN in the isolated heart. Here we show that NR affected cardiac IR injury similarly as reported for NMN: it protected hearts perfused with glucose and fatty acids, and protection was associated with glycolysis activation. Although NR was used at lower dosage in our study than NMN in the study by Nadtochij et al. [34] (0.17 mM NR versus 1 mM NMN for 20–25 min administration), the increase in NAD^+^ was larger for NR (two times increase) than for NMN treatment (1.4 times increase), supportive of the observation that NR has a higher bioavailability than NMN [48]. Glycolysis activation is an established and primary mechanism through which the heart can protect itself against IR injury, playing an important role in the cardioprotective actions of e.g., ischemic preconditioning, metformin, insulin, volatile anesthetics, adenosine, NO donors, Hypoxia-inducible factor 1-a (HIF1α) stabilizers and 5' adenosine monophosphate-activated protein kinase (AMPK) activators [58]. Glycolysis activation may protect against cell death through (1) facilitating glucose phosphorylation at mitochondrially bound hexokinase thereby lowering mitochondrial potential, mitochondrial ROS production, mitochondrial activation and protection against mitochondrial damage [7, 18, 45, 60], (2) facilitating ion pumps and exchanger at the plasma membrane knowing that these pumps mainly use glycolytically produced ATP [25], and (3) maintaining a low pH during early reperfusion thereby inhibiting opening of the mPTP [20]. In contrast to protection against cell death, increased glycolysis relative to glucose oxidation may be detrimental for the recovery of mechanical function of the heart [30]. However, this was not observed in the current study. It should thereby also be realized that the cellular mechanisms determining recovery of cardiac function after IR deviate from those cellular mechanisms dictating cell death, explaining the sometimes observed dichotomy between recovery of function and cell death after cardiac IR [37, 47]. Further experiments with blocked glycolysis in the presence of glucose and fatty acids are necessary to consolidate that NR’s protection is indeed through glycolysis activation, although the loss of protection in hearts without glycolysis or hearts with highly activated glycolysis already indicate a cause-effect relationship between NR and glycolysis activation for protection.

### Pre-ischemic insulin does not reduce IR injury and blocks protection by NR

The addition of insulin was not associated with reduced cardiac IR injury in the current study, which could have been anticipated knowing that insulin is a strong activator of glycolysis in the heart and can activate the reperfusion injury salvage kinase (RISK) and signal transducer and activator of transcription 3 (STAT3) pathway that are reported to be cardioprotective [15, 26]. However, in the studies showing cardioprotection by insulin, insulin was only administered at reperfusion. In the present study, insulin was already present at baseline, and previous studies from our laboratory have shown that insulin then loses its protective effects, likely through increased glycogen breakdown during ischemia resulting in detachment of hexokinase II from mitochondria due to accumulation of glucose-6-phosphate [49]. The amount of HKII bound to mitochondria at the end of ischemia and early reperfusion is a major determinant of cardiac IR injury [19, 36, 42]. Thus, although insulin activated aerobic glycolysis to a large extent (protective effect), it also activated anaerobic glycolysis during ischemia (detrimental effect) due to glycogen loading of the heart before ischemia, as reflected by the increased TOC in the insulin treated hearts. Apparently, the detrimental effects of extended anaerobic glycolysis during ischemia overruled the beneficial effects of an activated aerobic glycolysis during reperfusion with insulin present. That insulin can be protective is illustrated by the experiments were insulin is only administered at reperfusion, abolishing the detrimental effects of pre-ischemic insulin administration. NR was without any effect on TOC, indirectly suggesting that NR only activated aerobic, but not anaerobic, glycolysis, thereby preventing increased glycogen breakdown during ischemia with its detrimental effects. These findings are commensurate with no NR effects on lactate release in our model, whereby the release of lactate can be interpreted as a proxy for anaerobic glycolysis.

Maybe even more important was the observation that insulin abrogated protection by NR. This adds NAD^+^ precursors as cardioprotective agents to the growing list of cardioprotective interventions that loses protection in the presence of insulin. Fullmer et al. [16] were the first to report that insulin can nullify ischemic preconditioning, probably through pre-activation of the Akt signaling pathway. We recently demonstrated that insulin also abrogated protection by the specific NHE-1 inhibitor cariporide in the isolated mouse heart [49], which cannot be immediately explained by pre-activated Akt but more likely by the prolonged anaerobic glycolysis during ischemia facilitating extracellular acidosis and thereby loss of NHE-1 activity [49]. The most likely explanation for loss of NR protection is that with insulin glycolysis is already maximally activated such that increasing NAD^+^ cannot further activate glycolysis and protection is lost. Therefore, insulin may be an important factor to be considered in the now-ongoing quest to improve cardioprotective translation from preclinical animal models to the clinic.

### NR and the pentose phosphate pathway

One of the consistent findings of acute NR treatment of isolated hearts were the increased levels of the PPP intermediates R5P and S7P, independent of the glycolysis rate in the heart. Surprisingly, we were unable to find similar observations in the literature, indicating that this may be a novel finding for NAD^+^ precursors. Possible mechanisms may be related to NAD^+^ being phosphorylated into NADP^+^ by NAD^+^ kinases including nicotinamide nucleotide transhydrogenase (NNT) and NAD kinases (NADKs) [6, 13]. The NADP^+^ then feeds the 6-phosphogluconate dehydrogenase (6PGD) enzyme in the PPP to activate synthesis of R5P. In the current isolated mouse heart study, we specifically had chosen the C57BL/6N strain, knowing that this strain does contain the NNT enzyme, whereas the much more used C57BL/6J strain does not [46]. Alternatively, it is also possible that R5P is generated through the NADase CD38 converting the NR-generated NMN into R5P and nicotinamide [4, 24]. As recently reported by Flam et al. [12], there is a significantly drop of R5P and S7P in the failing human hearts with nonischemic dilated cardiomyopathy. This suggests that NR might be protective in patients with end-stage heart failure, further research might be fruitful to test it.

The activation of PPP can induce protection against cardiac IR injury through the generation of NADPH that can be used by the ROS-detoxifying systems thioredoxin (Trx) and glutathione (GSH) [6, 13]. However, since PPP intermediates were increased in both low glycolysis and high glycolysis hearts, and NR only protected the low glycolysis hearts, NR protection against cardiac IR injury through PPP activation seems unlikely. Finally, when examining how much ^13^C-glucose label during only 4 min infusion went into S7P for the intact hearts, the difference between low glycolysis and high glycolysis hearts was striking: in the low glycolysis, non-insulin, hearts we were unable to detect any ^13^C-labeled S7P, whereas for the high glycolysis, insulin-treated hearts, almost 30% of S7P was labeled by ^13^C-glucose within 4 min perfusion of ^13^C-glucose. These data suggest that the glucose-dependent S7P synthesis pathway is highly insulin dependent in the heart. Further research is warranted to examine this novel observation for PPP regulation by insulin within hearts.

### NR impairs energy status in presence of insulin

NR treatment of high glycolysis, insulin-treated, hearts was unable to activate aerobic glycolysis. On the contrary, there seems to be a detrimental effects of NR treatment on energy metabolic pathway regulation in these hearts. NR decreased anaerobic glycolysis as was reflected by the observed decreased lactate release. Insulin-treated control hearts increased lactate release almost 8 times, which is most likely due to increased glucose uptake by the heart. NR treatment in insulin-treated, high glycolysis hearts also demonstrated an increase in AMP content of the heart, an effect not observed in the low glycolysis hearts. Increases in cardiac AMP levels are generally considered as a sign of developing energy deficiency, which is supported by the lowering of ATP with NR in these hearts. However, NR was without effect on cardiac performance during the 25 min baseline perfusion (results not shown). One possible explanation for the decrease in ATP and the consequential increase in AMP is the observed inhibition of anaerobic glycolysis in these hearts with highly activated glycolysis and impaired fatty acid oxidation due to insulin, although glycolysis commonly only provides about 5% of the cardiac ATP synthesis [44]. NR in insulin-treated hearts was also associated with non-significant trends of decreased labeling of TCA and PPP intermediates*.* Alternatively, the NAD^+^ synthesis from NR through the NR kinase (NRK) will also consume ATP and may explain part of the ATP decrease in the heart. In summary, it seems that the addition of NR to insulin-treated hearts induce a brake on specific metabolic/energetic pathways that are currently unexplained. Further research is warranted to explore this phenemenon.

### Strength/weaknesses

We employed a one-pass perfusion system of the isolated heart to prevent recirculating substrates from the heart (e.g. lactate) to build up in the perfusate thereby creating uncertainty concerning the inflow of substrates to the heart over time. To reduce costs, we lowered albumin to 1% as compared to the 4% normally present in the blood. Albumin-bound fatty acids in the blood normally fluctuate between 0.2 mM, immediately after feeding, and 0.8 mM, at early reperfusion or in the fasting state [51, 59, 61]. Some studies have reported albumin-bound FA levels > 1.0 mM with reperfusion, but these high values were likely an overestimation due to not taking into account on-going lipolysis in heparin-treated blood in the test tube [51]. Fatty acid uptake into cells is in the end determined by the amount of free fatty acids (FFA), that is in equilibrium and determined by the ratio of FA bound to albumin. As long as this FA/albumin ratio is < 2, and in our study the ratio equals 1.2, the amount of FFA is linearly related to this ratio [39]. The ratio of FA to albumin in our perfusate, 0.2 mM FA to 1% (0.17 mM) albumin, is equal to the ratio reported at early reperfusion [59, 61], i.e. 0.8 mM FA to 4% albumin. Thus, the amount of FFA in our perfusate likely mimics the conditions of in vivo reperfusion levels. A similar concentration of 0.2 mM FA bound to 1% albumin have been previously used in other isolated mouse heart experiments [34].

The present study was set up to determine NR effects on pre-ischemic cardiac metabolism employing three different metabolic conditions. The study was able to show that NR activated glycolysis and that this activation associated with NR’s protection against IR injury. However, the study did not examine NR’s effect on cardiac metabolism during the ischemic period or the reperfusion period under different metabolic conditions. Further new studies will be needed to examine whether glycolysis activation by NR at baseline is also present during these periods that are of relevance to cardiac IR injury. It should also be realized that our short ^13^C-glucose administration protocol does not allow to estimate the contribution of glycogenolysis to glycolysis. However, since we only determined glycolysis during baseline conditions, i.e. before ischemia, previous work has demonstrated that glycolysis is then largely independent of glycogen turnover [14, 17, 27]. Finally, the increased glycolysis activity with NR in the (glucose + FFA) group, detected by the increased amount of ^13^C-enriched glycolytic intermediates within the 4-min ^13^C glucose perfusions, reflects increased flux through the glycolysis pathway and should not be confused with, and is not similar to, an increased absolute build-up of pathway intermediates. An increase in the absolute amounts of glycolytic intermediates can actually be detrimental to ischemic injury.

In our experiments, we have used the time of contracture (TOC) during ischemia as a proxy of the degree of anaerobic glycolysis during ischemia. This is based on previous research showing that contracture starts to develop when glycolysis stops in isolated ischemic hearts [28]. However, at that same time the free energy of ATP hydrolysis, the ΔG_ATP_, also falls below a critical level to support the ATPase activity of ion pumps and cross-bridge cycling and calcium slowly starts to rise [11]. Thus, it should be realized that it is not only the halting of anaerobic glycolysis that determines TOC but also other parameters (e.g. Ca^2+^), such that using TOC as proxy of anaerobic glycolysis during ischemia is not completely unambiguous.

### Translational potential

NAD^+^ precursors are now in various clinical trials for conditions such as heart failure, ageing, mitochondrial myopathy, Parkinson and brain function. Because the US FDA has issued a ban on the sale of dietary supplements containing NMN, NR is the only currently commercially available NAD supplement. In the present study, we demonstrated that cardiac NAD increased between 40 and 100% with a 25 min continuous supply of 50mg/L NR. A previous in vivo mice study showed that such increases in cardiac NAD can also be obtained 30 min after administrating of one dose of 500 mg/kg NMN intraperitoneal [54]. Another study showed a 30–40% increase in cardiac NAD following 4 weeks of oral gavage of 400mg/kg/d NR in diabetic mice [22]. Assuming an allometric scaling factor going from mice to humans of about 0.06–0.08 [35], this would translate into 2 g/day NR for human with an averaged body weight of 70 kg. This is actually exactly the dose examined in one of the more recent human trials concerning safety of NR supplementation [52], and which dosage is now examined for the treatment of human heart failure.

Here, we demonstrated that NR may possibly be used as a protectant in conditions of elective surgery with a temporary obstruction of blood flow to an organ or tissue, thereby causing ischemia–reperfusion injury. Our work suggest that NR may especially offer protection through activation of glycolysis in conditions of low insulin, but that its protective potential can diminish in the presence of insulin.

## Conclusions

In conclusion, the results demonstrate that NR treatment can acutely increase the NAD^+^ content of the isolated heart, indicating that NR is suitable as reperfusion therapy to raise cardiac NAD^+^ acutely independent of the metabolic milieu present. However, to offer protection against cardiac IR injury, NR needs to be able to increase glycolysis, demonstrating the necessity of glycolysis for NR’s protection. When glycolysis is already fully activated by high plasma levels of insulin, it is anticipated that NR’s protective effect against cardiac IR injury will be diminished or even absent. The study indicates that the perioperative metabolic condition (e.g. fasting, low insulin versus non-fasting high insulin conditions) likely dictates the efficacy of NR treatment as therapy against cardiac IR injury.

### Supplementary Information

Below is the link to the electronic supplementary material.

Supplementary file1 (DOCX 1041 KB)

## Data Availability

The authors declare that the datasets used and/or analyzed in the current study are available from the corresponding author upon reasonable request.

## Abbreviations

NAD: Nicotinamide adenine dinucleotide
IR: Ischemia–reperfusion
NR: Nicotinamide riboside
FA: Fatty acids
LDH: Lactate dehydrogenase
PEP: Phosphoenolpyruvate
PPP: Pentose phosphate pathway
R5P: Ribose-5P
S7P: Sedoheptulose-7P
TCA: Tricarboxylic acid
