# Supplementary material for: Insulin and glycolysis dependency of cardioprotection by nicotinamide riboside
Source: Basic Res Cardiol. 2024 Mar 25;119(3):403–18. doi: 10.1007/s00395-024-01042-4 (PMC11142987; doi:10.1007/s00395-024-01042-4)
Supplement: Supplementary file 1 — Supplementary file1 (DOCX 1041 KB) [file 395_2024_1042_MOESM1_ESM.docx]

**Supplement**

**Insulin and Glycolysis Dependency of Cardioprotection**

**by Nicotinamide Riboside**

Xiao Y^1,2^, Zhang H^1,2^, Wang Q^1,2^, Nederlof R^3^, Bakker D^1,2^, Siadari BA^1^, Wesselink MW^1^, Preckel B^1,2^, Weber NC^1,2^, Hollmann MW^1,2^, Schomakers BV^4,5^, van Weeghel M^2,4,5,6^, Zuurbier CJ^1,2^

^1^ Amsterdam UMC, University of Amsterdam, Laboratory of Experimental Intensive Care and Anesthesiology, Department of Anesthesiology, Meibergdreef 9, Amsterdam, The Netherlands

^2^ Amsterdam Cardiovascular Sciences institute, Amsterdam, The Netherlands

^3^ Institut für Herz- und Kreislaufphysiologie, Medizinische Fakultät und Universitätsklinikum Düsseldorf, Heinrich- Heine- Universität Düsseldorf, Düsseldorf, Germany

^4^ Laboratory Genetic Metabolic Diseases, Amsterdam University Medical Centers, location Academic Medical Center, University of Amsterdam, Amsterdam, the Netherlands

^5^ Core Facility Metabolomics, Amsterdam University Medical Centers, location Academic Medical Center, University of Amsterdam, Amsterdam, the Netherlands

^6^ Amsterdam Gastroenterology Endocrinology and Metabolism institute, Amsterdam, The Netherlands

Address for correspondence:

Coert J Zuurbier

Department of Anesthesiology, Amsterdam UMC

Meibergdreef 9, 1105 AZ

Amsterdam, The Netherlands

Phone: +31 (0) 205665259

Email: c.j.zuurbier@amsterdamumc.nl

Supplement Includes:

- **Supple Table.1-2**
- **Supple Fig.1-4**
- **Supple File 1-3 (individual file): Original metabolomics data**

**Table 1** Baseline characteristics for Langendorff perfused heart subjected to IR injury

|  | **Glu + FFA** | | **Lac + Pyr + FFA** | | | **Glu + FFA + insulin** | | |  |
| --- | --- | --- | --- | --- | --- | --- | --- | --- | --- |
|  | **Control** | **NR** | | **Control** | **NR** | | **Control** | **NR** | |
| **EDP(mmHg)** | 4.0±0.8 | 3.9±0.7 | | 3.9±0.8 | 4.0±0.8 | | 4.1±0.6 | 4.2±0.6 | |
| **DLVP(mmHg)** | 99±5 | 96±8 | | 77±9 | 76±4 | | 97±21 | 100±16 | |
| **HR (b.p.m)** | 358±25 | 368±62 | | 382±48 | 393±56 | | 362±36 | 376±46 | |
| **RPP(DLVP*HR)** | 35341  ±2958 | 35248  ±5453 | | 29787  ±6307 | 30046  ±5714 | | 34800  ±7153 | 37194  ±4678 | |
| **Body weight(g)** | 24.3±1.5 | 25.1±2.2 | | 26.3±1.7 | 25.3±1.9 | | 24.3±1.2 | 24.9±1.9 | |
| **Flow (ml/min/GWW)** | 13.1±1.2 | 12.8±1.2 | | 12.9±1.9 | 12.5±1.5 | | 13.6±2.1 | 12.5±1.0 | |
| **T (°C)** | 36.9±0.1 | 36.9±0.1 | | 36.8±0.1 | 36.8±0.1 | | 36.8±0.1 | 36.9±0.1 | |
| **n** | 8 | 8 | | 8 | 8 | | 9 | 8 | |

No differences were observed between groups within each series across all the baseline parameters. EDP: end diastolic pressure; DLVP: developed lefty ventricular pressure; HR: heart rate; RPP: rate pressure product; GWW: heart wet weight in grams; n: sample size. Data are presented as mean ± SD, normally distributed data was analyzed by independent t-test, non-normally distributed data was analyzed by Mann-Whitney U test.

**Table 2** Baseline characteristics for Langendorff- perfused hearts subjected to LC-MS measurements

|  | **Glu + FFA** | | **Lac + Pyr + FFA** | | **Glu + FFA + Insulin** | |
| --- | --- | --- | --- | --- | --- | --- |
|  | **Control** | **NR** | **Control** | **NR** | **Control** | **NR** |
| **EDP(mmHg)** | 3.8±0.5 | 3.8±0.4 | 6.0±0.8 | 6.5±0.9 | 4.5±0.6 | 4.4±0.5 |
| **DLVP(mmHg)** | 97±17 | 99±13 | 82±9 | 79±7 | 106±12 | 104±23 |
| **HR (b.p.m)** | 383±47 | 391±75 | 385±38 | 370±46 | 369±63 | 382±71 |
| **RPP(DLVP*HR)** | 36441  ±5272 | 38688  ±8126 | 31662  ±5239 | 29084  ±4872 | 39295  ±9554 | 38830  ±6465 |
| **Body weight(g)** | 25.7±2.5 | 26.5±2.3 | 24.6±2.1 | 24.7±0.8 | 23.7±1.3 | 24.3±0.9 |
| **T (°C)** | 36.7±0.1 | 36.7±0.1 | 36.7±0.1 | 36.7±0.1 | 36.8±0.1 | 36.8±0.1 |
| **n** | 8 | 8 | 9 | 8 | 8 | 8 |

No differences were observed between groups within each series across all the baseline parameters. EDP: end diastolic pressure; DLVP: developed lefty ventricular pressure; HR: heart rate; RPP: rate pressure product; n: sample size. Data are presented as mean ± SD, normally distributed data was analyzed by independent t-test, non-normally distributed data was analyzed by Mann-Whitney U test.

**
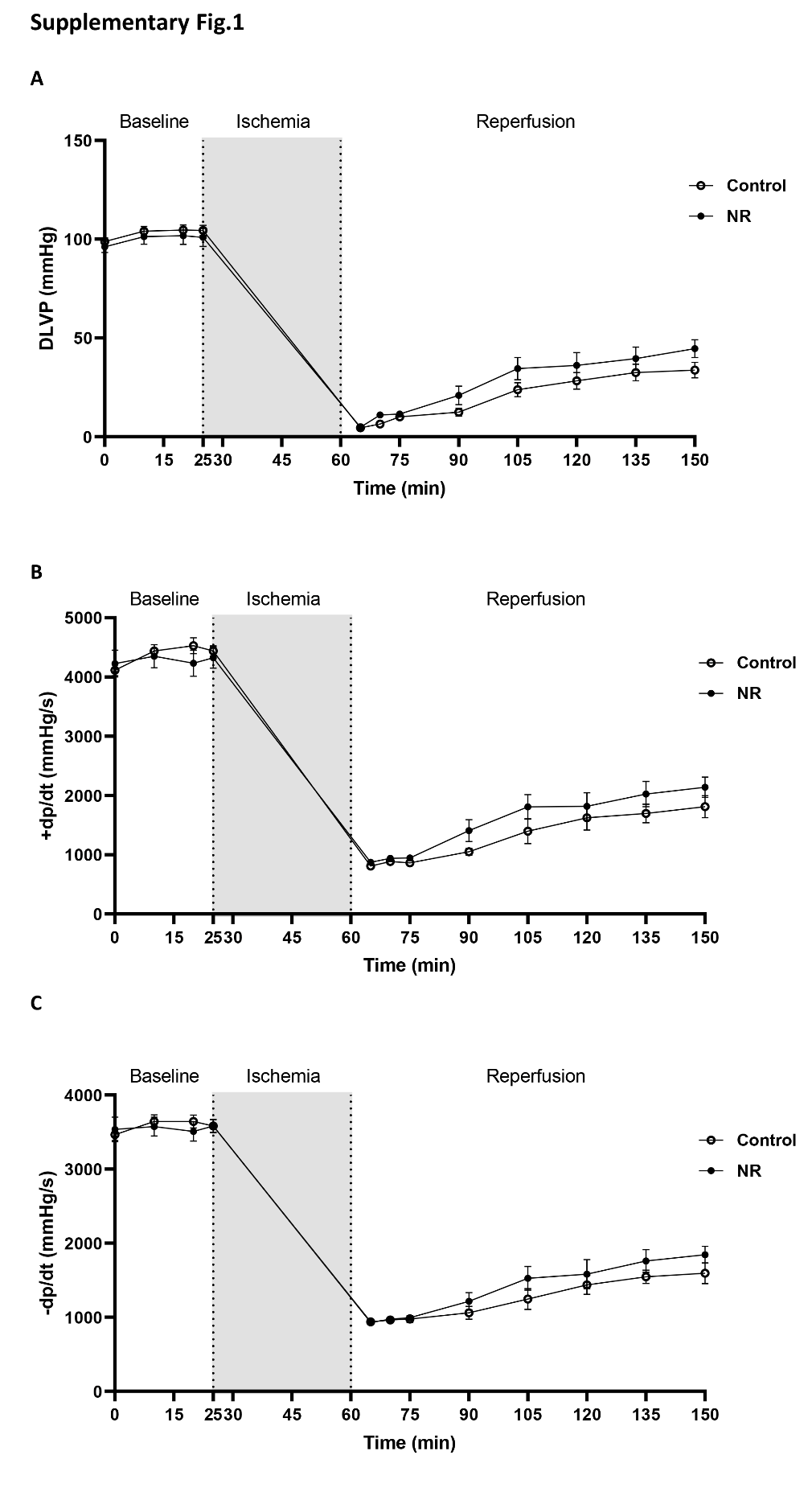
**

**Supplementary Fig.1** Hearts were perfused with glucose and free fatty acid (Glu+FFA). Line plots showing the time course for (A) developed left ventricular pressure (DLVP); (B) maximum contraction rate of left ventricle (+dp/dt); and (C) maximum relaxation rate of left ventricle (-dp/dt). Data are presented as mean ± SEM.

**
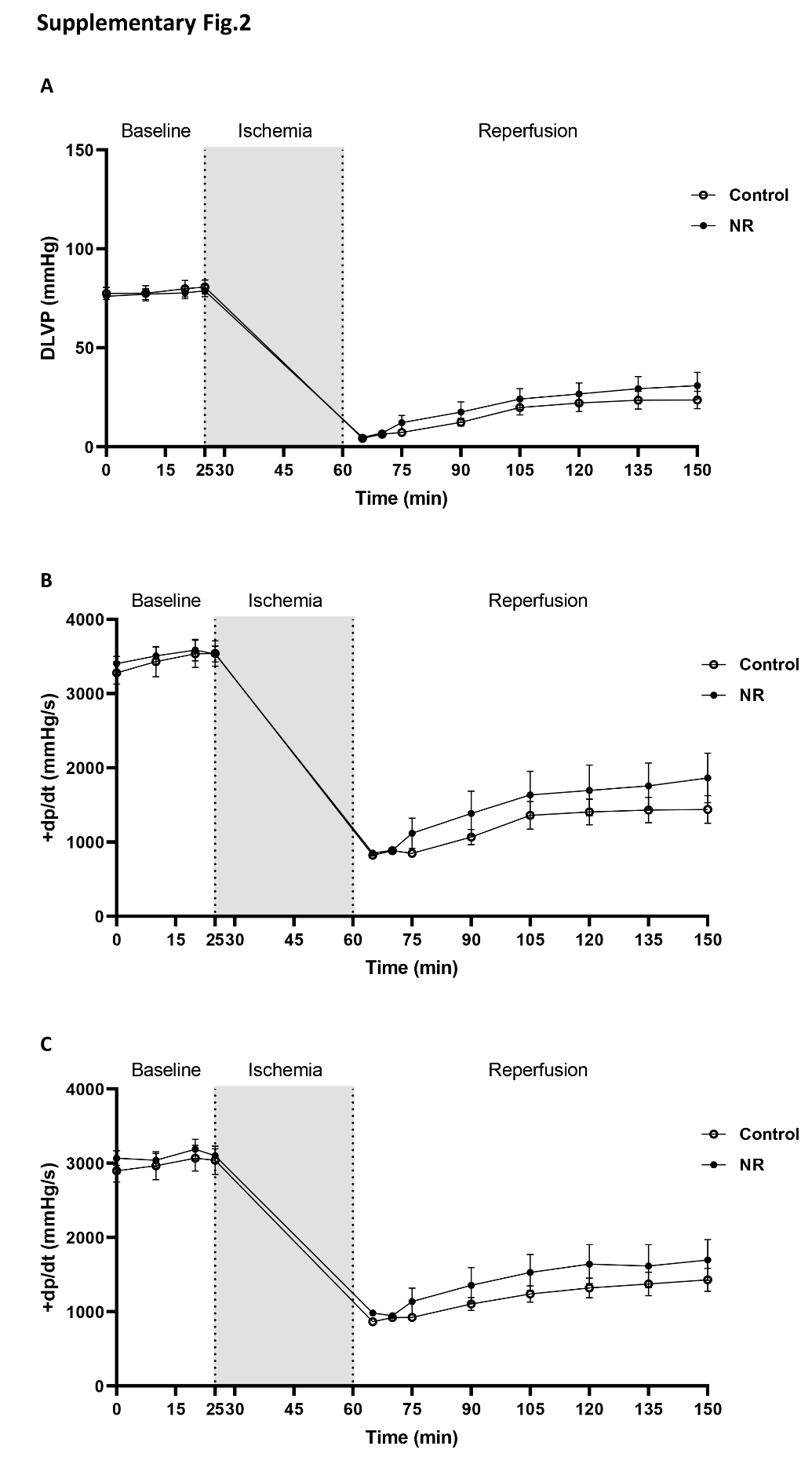
**

**Supplementary Fig.2** Hearts were perfused with lactate, pyruvate, and free fatty acid (Lac+Pyr+FFA). Line plots showing the time course for (A) developed left ventricular pressure (DLVP); (B) maximum contraction rate of left ventricle (+dp/dt); and (C) maximum relaxation rate of left ventricle (-dp/dt). Data are presented as mean ± SEM.


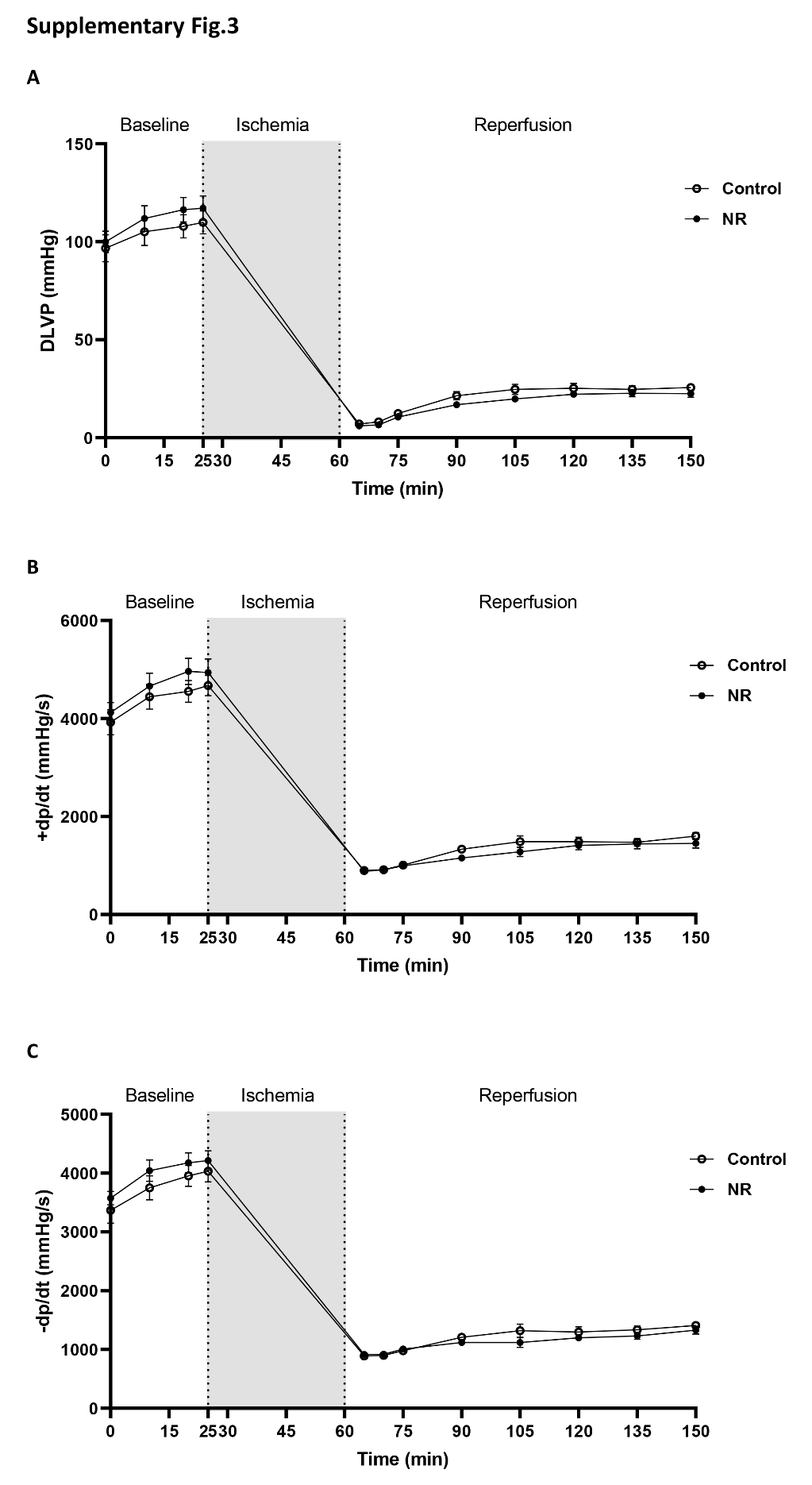


**Supplementary Fig.3** Hearts were perfused with glucose, free fatty acid, and insulin (Glu+FFA+Ins). Line plots showing the time course for (A) developed left ventricular pressure (DLVP); (B) maximum contraction rate of left ventricle (+dp/dt); and (C) maximum relaxation rate of left ventricle (-dp/dt). Data are presented as mean ± SEM.

**
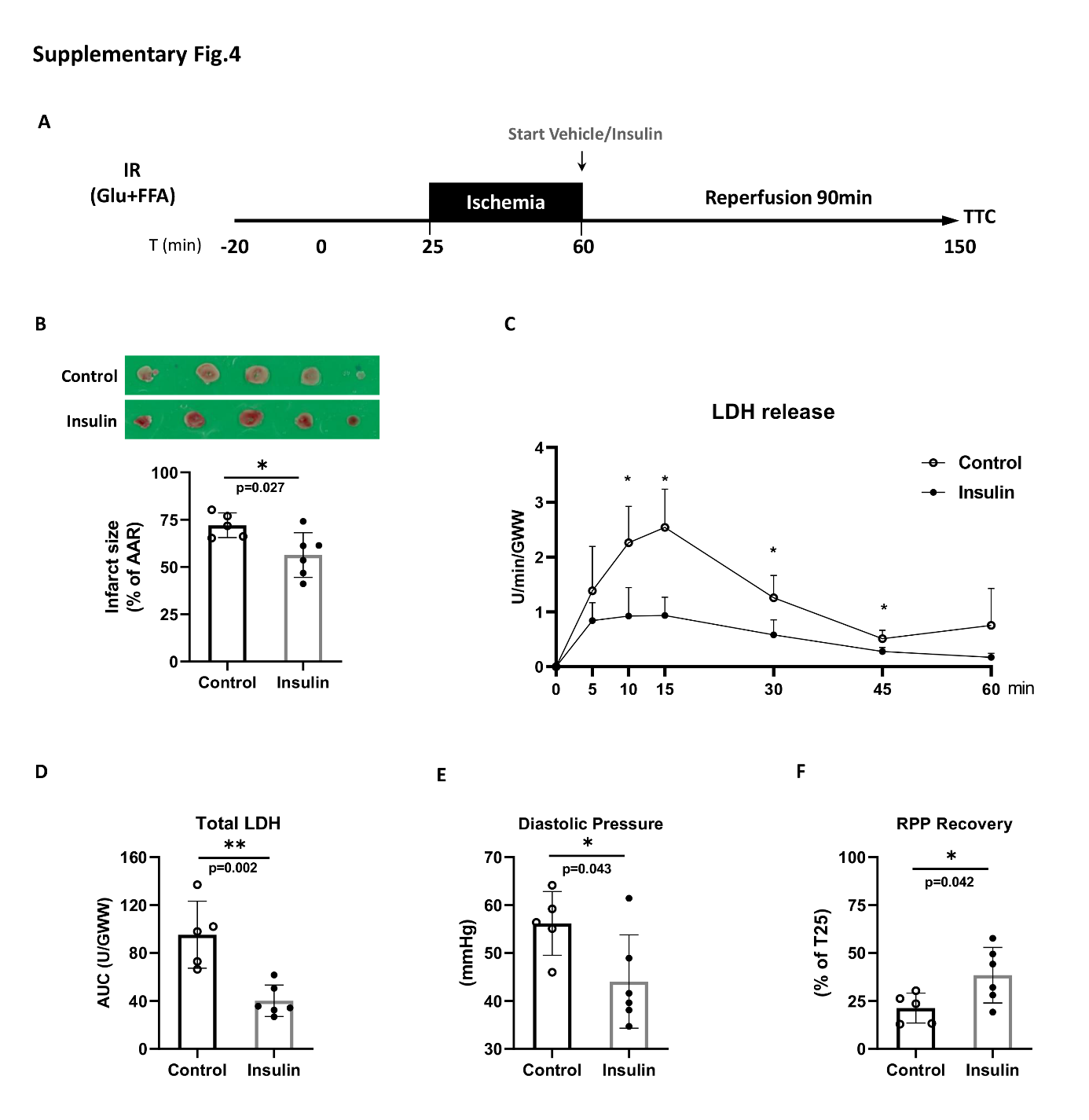
**

**Supplementary Fig.4** **Insulin administration only at reperfusion is cardioprotective**

(A) Hearts were perfused with glucose and free fatty acid (Glu+FFA). After 25min baseline, hearts were subjected to 35 min ischemia followed by 90 min reperfusion, insulin or vehicle was administrated only during the 90min reperfusion; (B) Image of TTC staining (upper panel) and quantified infarct size related to AAR (lower panel); (C) LDH release at different time points during reperfusion, normalized to coronary flow and heart wet weight; (D) Total LDH release during reperfusion, normalized to coronary flow and heart wet weight; (E) Diastolic pressure at the end of reperfusion; (F) RPP recovery at the end of reperfusion relative to baseline T= 25 min. **P*<0.05, ***P*<0.01 vs Control group.
